# Novel Outbreak-Associated Food Vehicles, United States

**DOI:** 10.3201/eid2710.204080

**Published:** 2021-10

**Authors:** Hilary K. Whitham, Preethi Sundararaman, Daniel Dewey-Mattia, Karunya Manikonda, Katherine E. Marshall, Patricia M. Griffin, Brigette L. Gleason, Sanjana Subramhanya, Samuel J. Crowe

**Affiliations:** Centers for Disease Control and Prevention, Atlanta, Georgia, USA (H.K. Whitham, P. Sundararaman, D. Dewey-Mattia, K. Manikonda, K.E. Marshall, P.M Griffin, B.L. Gleason, S. Subramhanya, S.J. Crowe);; Oak Ridge Institute for Science and Education, Oak Ridge, Tennessee, USA (S. Subramhanya)

**Keywords:** Foodborne diseases, disease outbreaks, enteric infections, novel food vehicles, prevention, food safety, United States

## Abstract

Novel outbreak-associated food vehicles (i.e., foods not implicated in past outbreaks) can emerge as a result of evolving pathogens and changing consumption trends. To identify these foods, we examined data from the Centers for Disease Control and Prevention Foodborne Disease Outbreak Surveillance System and found 14,216 reported outbreaks with information on implicated foods. We compared foods implicated in outbreaks during 2007–2016 with those implicated in outbreaks during 1973–2006. We identified 28 novel food vehicles, of which the most common types were fish, nuts, fruits, and vegetables; one third were imported. Compared with other outbreaks, those associated with novel food vehicles were more likely to involve illnesses in multiple states and food recalls and were larger in terms of cases, hospitalizations, and deaths. Two thirds of novel foods did not require cooking after purchase. Prevention efforts targeting novel foods cannot rely solely on consumer education but require industry preventive measures.

Foodborne illness is a major public health issue in the United States; millions of persons become ill from contaminated food every year (*1*). Most cases are sporadic (i.e., not associated with a disease outbreak) (*2*), and the responsible food(s) is often undetermined. Outbreaks provide an opportunity for public health agencies to determine shared exposures and the source of infection. Many food safety laws and regulations, industry practices, and consumer education efforts have been implemented to make foods safer. Nevertheless, evolving foodborne pathogens and changing consumption trends provide continued opportunities for contamination and illness (*3*–*7*). Within these changing conditions, novel outbreak-associated food vehicles (i.e., foods not implicated in prior outbreaks) can emerge. Identifying these novel food vehicles provides an opportunity to determine emerging sources of illness and to inform prevention policies. To identify novel food vehicles reported during 2007–2016, we examined data from the Centers for Disease Control and Prevention (CDC) Foodborne Disease Outbreak Surveillance System (FDOSS).

## Methods

FDOSS is a passive surveillance system that collects reports of foodborne disease outbreaks from federal, state, local, and territorial health departments in the United States. It is the primary source of data for outbreak-associated illnesses, hospitalizations, and deaths; etiologic agents; implicated foods; contributing factors; and preparation and consumption settings. Foodborne outbreaks are nationally notifiable and defined as ≥2 cases of a similar illness resulting from ingestion of the same food (*8*). When exposure occurs in 1 state, the outbreak is classified as a single-state outbreak; when exposure occurs in ≥2 states, the outbreak is classified as a multistate outbreak. Foods or specific ingredients are identified as sources by using epidemiologic, laboratory, traceback, and environmental assessment data. On the basis of this evidence, a sole food (e.g., apple) or a specific ingredient that is part of a complex food (e.g., beef in a sandwich) is reported as the source of an outbreak. A complex food is reported as the source when no specific ingredient is implicated. When an investigation does not identify a source, the food vehicle is reported as undetermined. CDC uses a hierarchical scheme to categorize reported foods (*9*,*10*). For simplicity and ease of interpretation, for this analysis we collapsed some categories (e.g., seeded and row crop vegetables are reported generically as vegetables).

Using FDOSS data from 1973–2016 (accessed December 11, 2017), we reviewed all 14,216 reported outbreaks with an implicated food or ingredient (henceforth, collectively referred to as food). We compared reported foods from outbreaks with a year of first illness onset during 2007–2016 with those during 1973–2006 by using a 3-stage process to identify novel outbreak-associated food vehicles. First, foods that were reported identically in both time frames were identified and removed by using SAS version 9.4 (SAS Institute Inc., https://www.sas.com). Second, 2 independent reviewers manually compared the remaining 878 food items and flagged any foods that seemed to be novel in 2007–2016. Third, a 5-member panel reviewed all foods initially identified as novel. For the second and third steps, we excluded foods if they were reported in a prior outbreak by using a different term (e.g., rice, wild vs. wild rice), a more general term (e.g., cheddar vs. white cheddar), or a different spelling or an abbreviation (e.g., brat vs. bratwurst) or if a specific contaminated ingredient(s) was not implicated for a newly reported complex food. We adopted this final exclusion criterion when reviewing complex foods in which no specific ingredient was implicated because it could not be determined what ingredients were included in the food itself, much less which ingredient was actually contaminated and could be novel (i.e., direct comparison between 1973–2006 and 2007–2016 was impossible).

We then conducted a secondary check of additional sources for all foods initially identified as novel (PubMed, online forums [e.g., Food Safety News, Food Poison Journal, and MarlerClark], and media reports). This check served to identify false-positive results from 2 scenarios: 1) the food had been implicated in an outbreak during 1973–2006, but the outbreak had not been reported to FDOSS; or 2) the food had been reported as part of an outbreak occurring during 1973–2006 with a more generic term. We reclassified foods only if the available information was sufficient to follow our criteria (i.e., there were >2 confirmed cases and an identified implicated food).

Novel food vehicles are presented along with key outbreak characteristics, including etiology and various measures of burden (e.g., case counts, deaths) in addition to statistical comparisons of these characteristics for outbreaks associated with food vehicles that are novel or not novel. Specifically, we used nonparametric Wilcoxon tests to assess difference in means and χ^2^ tests to assess differences in percentages. Statistical analyses were completed in R version 3.3.3 (R Foundation for Statistical Computing, https://www.r-project.org).

## Results

### Novel Food Vehicles

By comparing outbreaks from 2007–2016 with those from 1973–2006, we identified 28 novel food vehicles (Table 1); the most common were fish (6), nuts (6), fruits (4), vegetables (3), and meats (3). Two thirds of novel foods did not require cooking after they were purchased (e.g., blueberries, kale, various nuts), and half did not require refrigeration after purchase.

**Table 1:** Novel food vehicles implicated in outbreaks that occurred during 2007–2016, United States*

Food	Category	State (no. states)	Year of first illness	Etiology	No. illnesses	No. hosp.	No. deaths	Recall	Imported
Almaco jack	Fish	Florida	2014	Ciguatoxin	2	0	0	No	Yes
Apple†	Fruit	Multistate (12)	2014	*Listeria monocytogenes*	35	34	7	Yes	No
Bison	Meat	Tennessee	2008	STEC O157:H7	12	2	0	No	No
		Multistate (5)	2010	STEC O157:H7	10	NR	NR	Yes	No
Blueberries	Fruit	Multistate (6)	2009	*Salmonella enterica* serovar Muenchen	14	NR	0	No	No
		Minnesota	2010	*Salmonella* Newport	6	1	0	No	No
Carp	Fish	New York	2012	Other chemical or toxin, Haff disease‡	2	2	1	No	No
Cashews§	Nut/seed	Multistate (6)	2014	*Salmonella* Stanley	18	4	0	No	Yes
Chia seed¶	Nut/seed	Multistate (19)	2014	*Salmonella,* multiple serotypes#	45	7	0	Yes	Yes
Flour (wheat)	Grain	Multistate (24)	2015	STEC, multiple serogroups**	56	16	0	Yes	No
Frog††	Meat	Arizona	2015	*Salmonella* Javiana	5	1	0	No	No
Goose‡‡	Meat	New York	2013	*Campylobacter jejuni*	57	1	0	No	No
Hazelnuts	Nut/seed	Multistate (3)	2010	STEC O157:H7	8	3	0	Yes	No
		Multistate (2)	2016	*Salmonella* Typhimurium	6	1	0	Yes	No
Kale	Vegetable	Florida	2013	STEC O157:H7	7	5	0	No	No
		Wisconsin	2014	*Cryptosporidium parvum*	8	0	0	No	No
Lima beans	Vegetable	Florida	2009	Unknown	13	0	0	No	No
Lionfish	Fish	South Carolina	2013	Ciguatoxin	4	1	0	NR	NR
Mini peppers	Vegetable	Multistate (10)	2014	*Salmonella* Paratyphi B	21	5	0	No	Yes
Monchong	Fish	Hawaii	2013	Scombroid toxin	2	0	0	No	No
Moringa leaf¶	Herb/spice	Multistate (24)	2015	*Salmonella* Virchow	35	6	0	Yes	Yes
Papaya	Fruit	Multistate (25)	2011	*Salmonella* Agona	106	10	0	Yes	Yes
		Multistate (4)	2013	*Salmonella* Thompson	13	6	1	No	No
Pepper¶	Herb/spice	Multistate (4)	2008	*Salmonella* Rissen	87	NR	NR	Yes	No
		Multistate (45)	2009	*Salmonella* Montevideo	272	52	0	Yes	Yes
Pine nuts	Nut/seed	Multistate (6)	2011	*Salmonella* Enteritidis	53	2	0	Yes	Yes
Pistachios	Nut/seed	Multistate (21)	2008	*Salmonella*, multiple serovars§§	83	NR	0	Yes	No
		Multistate (6)	2013	*Salmonella* Senftenberg	8	1	0	Yes	No
		Multistate (9)	2016	*Salmonella*, multiple serovars¶¶	11	2	0	Yes	No
Pomegranate	Fruit	Multistate (10)	2013	Hepatitis A virus	157	70	0	No	Yes
Sheep milk##	Dairy	Multistate (14)	2012	*Listeria monocytogenes*	23	21	5	Yes	Yes
Skate	Fish	New York	2008	Scombroid toxin	3	0	0	No	No
Sprouted nut butter***	Nut/seed	Multistate (10)	2015	*Salmonella* Paratyphi B variant L(+) tartrate (+)	13	0	0	Yes	No
Sugar cane	Sugar	Multistate (3)	2013	*Salmonella* Virchow	7	1	0	No	Yes
Swai	Fish	New York	2014	Unknown	3	1	0	No	Yes
Tempeh†††	Grain	North Carolina	2012	*Salmonella* Paratyphi B var. L(+) tartrate (+)	89	8	0	Yes	No

*Data from Centers for Disease Control and Prevention Foodborne Disease Outbreak Surveillance System (FDOSS), 1973–2016. Five of the 6 outbreaks linked to fish were caused by naturally occurring toxins that cannot be destroyed through cooking or freezing. Hosp., hospitalization; NR, not reported; STEC, Shiga toxin–producing *Escherichia coli*. †Apples were the contaminated ingredient in an outbreak associated with caramel apples. Prior apple cider outbreaks have been reported to FDOSS with no specific ingredient identified. ‡Haff disease is a syndrome of unexplained rhabdomyolysis caused by consumption of an unidentified toxin (rhabdomyolysis is a clinical syndrome caused by injury to skeletal muscle that results in the release of muscle cell contents into the circulation). §Cashews were processed into raw cashew cheese. ¶Product was processed and sold as a ground powder. #*Salmonella* Gaminara, Harford, Oranienburg, and Newport **STEC O26:NM and O121 ††Frog legs were from a noncommercial source. ‡‡Goose liver was the implicated ingredient of foie gras. Prior foie gras outbreaks have been reported to FDOSS with either no specific ingredient identified or a different implicated ingredient. §§*Salmonella* Montevideo, Newport, and Senftenberg. ¶¶S*almonella* Senftenberg and Montevideo. ##Pasteurized sheep milk, the only dairy food vehicle identified, was used in making ricotta salata cheese, which was later contaminated. ***Multiple nut butters from 1 company were implicated (cashew, almond, and hazelnut). †††Tempeh was unpasteurized.

### Outbreaks Associated with Novel Food Vehicles

A total of 36 outbreaks were linked to the 28 novel food vehicles during 2007–2016, and 7 foods were implicated in >1 outbreak (bison meat, blueberries, hazelnuts, kale, papaya, pepper, and pistachios). These 36 outbreaks resulted in 1,294 illnesses, 263 hospitalizations, 14 deaths, and 17 recalls. An average of 3.6 (range 0–8) outbreaks associated with a novel food vehicle were reported each year. Among outbreaks linked to a novel food vehicle, 22 (61%) occurred in multiple states; the largest multistate outbreak resulted from ground pepper in salami, involving 45 states and 272 illnesses (*11*). Etiologies included bacteria (27 [75%] outbreaks), toxins (5 [14%]), viruses (1 [3%]), and parasites (1 [3%]). The most commonly reported etiologic agent was *Salmonella* (19 [53%] outbreaks), followed by Shiga toxin–producing *Escherichia coli* (5 [14%]). Among outbreaks linked to novel food vehicles, 33% resulted from foods imported from another country.

Outbreaks associated with novel food vehicles differed from other outbreaks (i.e., those not associated with a novel food vehicle) in several ways (Table 2). First, 61.1% of outbreaks associated with a novel food vehicle involved exposure in multiple states, compared with 5.7% of other outbreaks (p<0.001). Second, 48.6% of outbreaks associated with a novel food vehicle resulted in a food recall, compared with 5.2% of other outbreaks (p<0.001). Third, the mean numbers of reported primary cases, hospitalizations, and deaths were greater among outbreaks linked to novel food vehicles than among other outbreaks (p = 0.04, p<0.001, and p<0.001, respectively). Fourth, the percentage of cases that resulted in hospitalization and the percentage of cases that resulted in death were significantly greater among outbreaks linked to novel food vehicles than among other outbreaks. Last, outbreaks associated with a novel food vehicle were more likely than other outbreaks to be caused by *Salmonella* contamination (p<0.001). Two potential confounding effects were a disproportionate number of *Salmonella* outbreaks linked to novel foods and potential effects of contamination from ill food workers (sensitivity analyses in Table 2).

**Table 2:** Features of outbreaks associated with novel and other food vehicles, United States, 2007–2016*

Feature	Food vehicle type	p value†

Novel						Other				
Outbreaks		Statistic				Outbreaks		Statistic		
Mean	Median	Range	Mean	Median	Range
No. cases per outbreak												
Primary	36		35.9	13.0	2–272		3,722		21.4	9.0	2–1,939	0.04
Hospitalized	32		8.2	2.0	0–70		3,502		1.6	0.0	0–308	<0.001
Died	34		0.4	0.0	0–7		3,520		0	0.0	0–33	<0.001
% Cases per outbreak												
Hospitalized	32		25.4	16.9	0–100		3,502		9.9	0.0	0–100	<0.001
Died	34		2.9	0.0	0–50		3,520		0.4	0.0	0–100	<0.001
Outbreaks, no. (%)												
Multistate	36 (61.1)						3,722 (5.7)					<0.001
Had recall	35 (48.6)						3,567 (5.2)					<0.001
Etiology *Salmonella*‡	34 (55.9)						2,226 (28.3)					<0.001

*Data from Centers for Disease Control and Prevention Foodborne Disease Outbreak Surveillance System, 2007–2016. Analysis limited to outbreaks with an implicated food. This analysis included outbreaks resulting from a range of contributing factors, including contamination from ill food workers (and not resulting from more upstream processes). None of the outbreaks associated with a novel food vehicle were linked to an ill food worker. As a sensitivity analysis, 584 outbreaks linked to ill food workers were excluded from the comparison group, leaving 3,138 outbreaks. Among these, the median number of primary cases was 8.0; hospitalizations, percent of cases hospitalized, and deaths, and percent of cases resulting in death were all 0; 6.8% of outbreaks were multistate, 6.1% had a recall, and 31.6% had an etiology of *Salmonella*. All statistical results remained robust with p<0.05. †Nonparametric Wilcoxon testing was used to assess statistical difference in means. χ^2^ testing was used to assess statistical differences in percentages. ‡Limited to single-etiology outbreaks that met confirmation guidelines. Outbreaks associated with a novel food vehicle were more likely to be caused by *Salmonella* contamination. These outbreaks are more likely to result in large, multistate outbreaks leading to public health investigations. As a sensitivity analysis, we restricted the sample to outbreaks with an etiologic agent of *Salmonella* leaving 649 outbreaks (19 linked to novel food vehicles and 630 linked to other outbreaks). Case effects did not remain significant (i.e., when comparing novel and other outbreaks, we found no statistically significant differences in primary cases, hospitalization, deaths, as well as percent of cases hospitalized and percent of cases resulting in death). However, outbreaks associated with a novel food were more likely than other outbreaks to have cases exposed in multiple states (84.2% for novel and 17.8% for other outbreaks, p<0.001) and result in a recall (63.2% for novel and 8.9% for other outbreaks, p<0.001).

## Discussion

We identified 28 novel foods linked to outbreaks that occurred during 2007–2016 in the United States. Compared with other outbreaks, those linked to novel foods were more likely to involve illnesses in multiple states; result in a food recall; and to be associated with, on average, larger numbers of cases, hospitalizations, and deaths. Investigating large and complex outbreaks requires considerable government resources and major costs to the public and industry. Moreover, two thirds of novel outbreak-associated food vehicles did not require cooking after purchase, and roughly half of novel foods did not require refrigeration. These factors highlight the need for targeted industry efforts to reduce contamination before point of purchase to protect consumers.

Food consumption patterns are dynamic, influenced by dietary trends, public health messaging, food accessibility, advertising, and affordability (*6*,*7*). Increased consumption of a food results in more opportunities for exposure and potentially larger outbreaks. Importing of foods and beverages into the United States has increased; average annual growth in economic value from 2007–2017 was 5.9% (*12*). As a result, access has expanded to a broader range of foods from diverse areas. One third of outbreaks linked to novel foods resulted from imported foods, whereas the overall percentage of outbreaks reported to CDC with an imported food implicated is relatively small (5% during the period 2009–2014) (*13*).

Identifying of novel outbreak-associated foods highlights the need for improvements in public health. The 3 key areas are outbreak investigation, prevention, and communications.

First, identifying the source of an outbreak can be difficult. Especially in multistate outbreaks, investigators typically use standardized food history questionnaires to identify common foods among a sample of patients. Investigators develop questionnaires largely on the basis of trends in previous outbreaks and are influenced by common—rather than novel—food vehicles. However, identifying novel foods may require a detailed investigation of everything consumed by patients during the exposure period. A targeted questionnaire can be developed on the basis of data from these in-depth, hypothesis-generating interviews and administered to a larger group of patients and controls. Barriers to this approach include limited resources, reluctance of investigators to allow investigators from other areas to interview patients in their jurisdiction, lack of training with regard to conducting hypothesis-generating interviews, and the assumption that more interviews with the standardized questionnaire will eventually reveal the underlying food vehicle(s). Efforts to further engage, fund, and support states in investigating and reporting outbreaks are needed to effectively identify novel food vehicles.

Second, identifying novel food vehicles provides opportunities for new prevention measures. The occurrence of >2 outbreaks linked to novel foods (we identified 7 instances) may serve as a warning signal for public health authorities indicating gaps in food safety practices, regulatory oversight, or both. Targeted outreach to industry may be pursued when a novel food is identified. Focused, collaborative prevention efforts undertaken by industry in collaboration with academic institutions, regulatory agencies, consumer advocacy groups, and other nonprofit public health organizations have been successful in the past and should become standard practice.

Third, communications regarding novel food vehicles to the public, industry, and regulatory agencies could be improved. Intensive public health messaging may be needed to notify the public, industry, and public health partners of newly discovered risks to prevent additional illnesses. This type of communication is regularly performed by public health and regulatory agencies, but greater emphasis on the novelty of the food source could attract additional media attention and, in turn, lead to greater awareness.

The first limitation of our analysis is that it encompasses reported outbreaks with data on implicated foods representing both a subset of foodborne outbreaks occurring in the United States and of outbreaks reported to FDOSS. As a result, these novel outbreak-associated food vehicles may not actually be novel; rather, previous outbreaks associated with these vehicles may not have been detected or reported. The second limitation is that only a small percentage of foodborne illnesses are linked to recognized outbreaks (outbreak-associated cases are estimated to range from 0.5% of laboratory-diagnosed *Campylobacter* cases to 19.0% of Shiga toxin–producing *E. coli* O157 cases) (*2*), and it is possible that outbreak-associated novel foods differ from those linked to sporadic foodborne illnesses. The third limitation is the possibility that novel food vehicles were misclassified. Considerable efforts were made to reduce false-positive identifications by searching for different spellings, alternative names, and shared ingredients, as well as by using a team of independent reviewers. We also checked secondary sources for foods identified as novel from FDOSS data. This effort identified 2 food vehicles (mamey fruit and jalapeño peppers) that incorrectly initially seemed to be novel because of the use of more generic food terms in reports before 2007.

In summary, identifying novel food vehicles for foodborne illness provides opportunities for early interventions that should not go unheeded. This analysis highlights the need for expanded food safety measures to reduce opportunities for contamination as foods are grown, harvested, and processed and to prevent illness from both novel and other food sources in the United States and abroad. Greater investment in public health outbreak investigation capacity may increase and expedite identification of novel food vehicles, which is vital because novel foods may serve as signals for emerging threats.
